# Use of kidscreen 52 questionnaire for quality of life evaluation in adolescents with idiopathic scoliosis after surgery

**DOI:** 10.1186/1748-7161-8-S1-O58

**Published:** 2013-06-03

**Authors:** G Rusovs, O Audrupe, J Ositis, A Vetra

**Affiliations:** 1 Riga Stradins University, Riga, Latvia

## Background

Improvement of patients quality of life is one of the main goals in the treatment of AIS. There are well known instruments to estimate the treatment outcomes for patients with AIS, such as SRS questionnaires. Although widely used with patients with AIS, there are still some important aspects of QOL less covered in those instruments. The KIDSCREEN-52 quality-of-life (KIDSCREEN-52-HRQOL) is a relevant, worldwide tool used for assessing the health-related quality of life in children and adolescents, and to compare the QOL of treated patients with healthy peers at the same age.

## Aim

To describe and characterize health-related quality of life in girls with idiopathic scoliosis, after spine surgery, with the KIDSCREEN-52-HRQOL questionnaire.

## Methods

Patients with idiopathic scoliosis completed an age-appropriate health-related quality of life questionnaire KIDSCREEN-52 test approved at country the research provided. The study involved 50 patients with AIS. Patients were 12-16 years old girls, post surgery. The results from this sample were compared with general population norms, taken from freely chosen peers without any orthopedic condition.

## Results

The results where similar in the following blocks - ""feelings"", ""family and home"", ""leisure"", ""school"" and a ""bad mood"". AIS group had a higher scores at blokes as ""to be happy as you are"" ""feeling happy at home"" , ""feel satisfied with life"" and ""happy to be alive"". In the question blocks as ""physical activity"", ""Self esteem” and “Money” AIS patients revealed lower results with statistically significant difference 24.8% (t = 13.237, p = 0.001), 16.3% (t = 8.923, p = 0.001) and 22,8% (t = 7,522; p = 0,001) respectively.

## Conclusion

By using questionnaire KIDSCREEN 52, it is possible to compare HRQOL of AIS patients, after surgery, with the QOL of healthy peers, and reveal some aspects, not covered by widely used questionnaires, for estimation of treatment outcomes of AIS.
